# The Role of Heat Shock Protein 70 Subfamily in the Hyperplastic Prostate: From Molecular Mechanisms to Therapeutic Opportunities

**DOI:** 10.3390/cells11132052

**Published:** 2022-06-28

**Authors:** Xun Fu, Huan Liu, Jiang Liu, Michael E. DiSanto, Xinhua Zhang

**Affiliations:** 1 Department of Urology, Zhongnan Hospital of Wuhan University, Wuhan 430000, China; 2015302180157@whu.edu.cn (X.F.); henryliumed@foxmail.com (H.L.); zijiang.qing@gmail.com (J.L.); 2 Department of Surgery and Biomedical Sciences, Cooper Medical School of Rowan University, Camden, NJ 08028, USA; disantom@rowan.edu

**Keywords:** BPH, LUTS, HSP70 subfamily, HSP70s, targeted therapies

## Abstract

Benign prostatic hyperplasia (BPH) is one of the most common causes of lower urinary tract symptoms (LUTS) in men, which is characterized by a noncancerous enlargement of the prostate. BPH troubles the vast majority of aging men worldwide; however, the pathogenetic factors of BPH have not been completely identified. The heat shock protein 70 (HSP70) subfamily, which mainly includes HSP70, glucose-regulated protein 78 (GRP78) and GRP75, plays a crucial role in maintaining cellular homeostasis. HSP70s are overexpressed in the course of BPH and involved in a variety of biological processes, such as cell survival and proliferation, cell apoptosis, epithelial/mesenchymal transition (EMT) and fibrosis, contributing to the development and progress of prostate diseases. These chaperone proteins also participate in oxidative stress, a cellular stress response that takes place under stress conditions. In addition, HSP70s can bind to the androgen receptor (AR) and act as a regulator of AR activity. This interaction of HSP70s with AR provides insight into the importance of the HSP70 chaperone family in BPH pathogenesis. In this review, we discuss the function of the HSP70 family in prostate glands and the role of HSP70s in the course of BPH. We also review the potential applications of HSP70s as biomarkers of prostate diseases for targeted therapies.

## 1. Introduction

Benign prostatic hyperplasia (BPH) is a ubiquitous chronic disease affecting elderly males worldwide [1]. Bothersome lower urinary tract symptoms (LUTS) are one of the most frequent clinical symptoms of BPH [2]. LUTS include a wide range of symptoms, such as urgency, nocturia, frequency, dysuria and difficulty emptying the bladder [3], all of which adversely affect the health and life quality of aging men. The incidence rate of BPH/LUTS increases with age [4]. BPH morbidity in men aged 60–69 is 70%, and it increases to 90% in men aged >80 years [3,5]. Likewise, LUTS affect 44% of males aged 40–59 years and 70% of males over 80 years of age [6,7]. Apart from age dependence and sex hormones (androgen and estrogen), the imbalance of androgen/estrogen ratio, the dysregulation of cell proliferation and apoptosis, the interaction between stromal and epithelial cells, inflammation, and growth factors are other accepted predisposing factors of BPH [8]. Recently, epithelial/mesenchymal transition (EMT), which promotes cancer cell invasion and metastasis, along with oxidative stress (OS) that occurs when cellular homeostasis is disrupted, has been reported to have a link with the initiation of BPH [9,10,11,12]. Despite the growing publication of mechanistic studies about this disease, its exact pathogenesis still remains unclear.

Heat shock proteins (HSPs) are molecular chaperones that maintain cellular homeostasis and respond to various forms of cell stress [13]. In response to numerous stress factors, HSPs are activated and overexpressed to exert their functions of promoting the correct folding of proteins and degrading misfolded proteins [13,14]. The HSP family is evolutionarily conserved and ubiquitously expressed in all organisms and various cellular compartments [15]. Based on molecular weight, HSPs are commonly classified into six subfamilies: HSPH (HSP110), HSPC (HSP90), HSPA (HSP70), DNAJ (HSP40), HSPB (small HSPs (sHSPs)), as well as chaperonin families: HSPD/E (HSP60/HSP10) and CCT (cytosolic chaperonin TCP1 ring complex (TRIC)) [16]. The members of the HSP70 subfamily weigh 68–78 kDa and primarily include HSP70, glucose-regulated protein 78 (GRP78) and mortalin [17]. It has been noted that HSP70s modulate multiple biological processes associated with the development of BPH, such as cell survival and proliferation, cell apoptosis and the androgen receptor (AR) pathway [16,18,19]. These molecules are also involved in EMT and OS, acting as key regulators in two processes [18,20]. In this review, we highlight the molecular role of the HSP70 subfamily in BPH pathogenesis. We also discuss the potential value of HSP70s as therapeutic targets in BPH treatment.

## 2. Overview of Benign Prostate Hyperplasia

Histologically, benign enlargement of the prostate gland in BPH patients is prostatic hyperplasia (increase in cell number) but not hypertrophy (increase in cell size). Both epithelial and stromal cells proliferate excessively during the development of BPH (the phase of pathological BPH) [21], but epithelial/stromal ratios are pleomorphic in resected prostate samples. A histological study in the late 1970s showed that some BPH nodules are purely glandular or stromal, and some are mixed [22]. This phenotypic heterogeneity in BPH has a significant impact on therapeutic efficacy and appears to be related to drug resistance [23]. A subset of men with prostate enlargement suffers from a wide range of symptoms of the urinary system (i.e., LUTS). This condition is commonly referred to as clinical BPH, a phase that impairs the life quality of patients and therefore requires corresponding treatment.

The pathogenetic factors of BPH are quite complex and have not been completely identified. One of the basic characteristics of this disease is abnormal prostatic growth caused by disruption of normal glandular homeostasis between cell proliferation and cell death [8]. Cell cycle machinery is of great importance in controlling cellular proliferation, and promotion of cell cycle progression leading to hyperproliferation of prostatic cells is one of the potential mechanisms responsible for prostatic hyperplasia. Cordon-Cardo et al. firstly reported that p27KIP1, a negative regulator of the cell cycle, decreased within hyperplastic prostate tissues, while lack of the p27KIP1 gene increased the proliferative activity of prostatic cells [24]. Our recently published evidence also suggested that bone morphogenetic protein 5 (BMP5) and Smoothened (SMO), alongside the downstream glioma-associated oncogene (GLI) family, stimulated cellular proliferation of BPH-1 and WPMY-1 cells by promoting cell cycle progression [25,26]. Apoptosis has been known as one of the programmed cell death mechanisms. Kyprianou et al. supposed the evasion from normal apoptotic machinery may be responsible for the prostatic overgrowth [27]. In addition to apoptosis, pyroptosis is another type of programmed cell death related to the development of BPH. Jiang et al. recently published an in vitro study providing evidence for the relationship between PRDX3, pyroptosis and BPH [28].

Oxidative stress, one of the predisposing factors of BPH, is characterized by disruption of homeostasis between production and elimination of oxidants, including but not limited to reactive oxygen species (ROS) [29]. The OS process results from overproduction of oxidants, reduced antioxidant activity or both and is reported to induce DNA damage (e.g., mutations, deletions or rearrangements) and reduce DNA repair, both of which eventually stimulate compensatory cellular proliferation and overgrowth of the prostate gland [30]. Higher levels of oxidants and oxidative products, including inducible nitric oxide (iNOS) and reactive nitric species [31], nitric oxide (NO) [32] and plasma peroxides [33], have been observed in BPH patients compared with controls. Inflammatory cells are the well-accepted main source of ROS in the course of BPH, while the HSP70 family was recently found to have a link with ROS generation. On the other hand, the impaired antioxidant system loses its ability to alleviate oxidative stress and therefore exacerbates ROS-induced damage to prostatic tissues. Olinski and colleagues found that the majority of BPH tissues showed a comparably lower activity of superoxide dismutase (SOD) and catalase (CAT) [34]. As a result, both overproduction of oxidants and decreased antioxidant activity are the causes of the occurrence of OS in the hyperplastic prostate.

The EMT process allows epithelial cells to lose their epithelial characteristics and acquire mesenchymal phenotypes, which provides novel insight into the origin of stromal cells, such as myofibroblasts and smooth muscle cells, in the hyperplastic prostate. Based on the characteristics of EMT, Paloma et al. speculated that enlargement of the prostate gland may result from the accumulation of cells as a result of epithelial proliferation and the EMT process [9] and this speculation was confirmed by lower expression of E-cadherin (an epithelial marker)and higher expression of vimentin (a mesenchymal marker) in hyperplastic tissues [9,35].

Early in the 1980s, comparably higher nuclear AR levels were observed in the hyperplastic prostate [36,37]. As a member of the nuclear hormone receptor superfamily, AR has only been detected in the nucleus of prostate cells, which was confirmed by data on both normal prostate tissues and hyperplastic prostate tissues [16]. Recently, AR has been found to modulate the cellular growth of both stromal and epithelial cells, as well as the EMT process [9,38]. In vivo modeling of AR knockout showed that loss of stromal AR decreased the proliferative activity of prostatic cells and the size of the anterior prostate lobes [39,40,41]. Stromal AR was also documented to stimulate stromal cell proliferation by recruiting infiltrated macrophages [42]. In contrast, loss of AR signaling in luminal cells increases the proliferative rate. Thus, the form of AR action seems strikingly different within epithelial and stromal cells. This concept is supported by a study reporting that androgen treatment increased AR activity in epithelial cells but decreased its activity in stromal cells [43]. In fact, AR binding site data from CHIPseq indicated a distinct difference in AR target genes within epithelial and stromal cells [44], which also provided evidence for the differences in the action form between epithelial AR and stromal AR. Furthermore, epithelial AR promotes BPH development via macrophage-mediated EMT, indicating that AR in BPH-1 and mPrE cells can recruit macrophages and enhance the EMT process [45]. In general, it is evident that the AR, either epithelial AR or stromal AR, is implicated in BPH pathogenesis. However, a number of mechanistic studies are still necessary to uncover the relationship between AR expression and BPH etiology.

## 3. Members of HSP70 Family

The HSP70 family contains 13 members; these members include but are not limited to inducible HSP70 (HSP72 or HSPA1), constitutive heat shock cognate protein 70 (HSC70), GRP78 (Bip or HSPA5) and mortalin (GRP75 or HSPA9) [46]. Genes encoding HSP70 proteins are widely expressed within various cellular compartments, including cytoplasm, nucleus/nucleoli and multiple organelles (e.g., ER, proteasomes, ribosomes, mitochondria and lysosomal membranes). HSP70 proteins also function on the cell surface, at cytoskeletal frameworks, and in the intercellular space.

**Figure 1:**
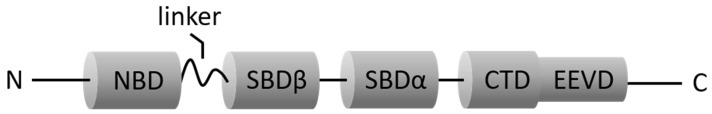
Schematic diagram demonstrating domain organization of HSP70 family. HSP70 family members are composed of highly conserved N-terminal nucleotide-binding domain (NBD) and C-terminal substrate-binding domain (SBD) that are connected by a linker. The latter domain is subdivided into β-sandwich subdomain (SBDβ) and α-helical subdomain (SBDα), which is followed by a disordered C-terminal domain (CTD) that frequently ends with a charged EEVD motif.

The HSP70 family is highly conserved in evolution. The molecular structure of HSP70s exhibits two common domains: an N-terminal nucleotide-binding domain (NBD) and a C-terminal substrate-binding protein domain (SBD), both of which are connected to each other by a linker (Figure 1). The NBD is further subdivided into four subdomains (IA, IB in lobes I and IIA, IIB in lobe II); between the two lobes exists a cleft that is the ATP-binding site. The SBD is sequentially divided into a β-sandwich subdomain (SBDβ) and α-helical subdomain (SBDα), followed by a disordered C-terminal tail of variable length (C-terminal domain (CTD)). Within cytosolic and nuclear HSP70s in eukaryotic organisms, the CTD commonly ends with a charged motif (Glu-Glu-Val-Asp; EEVD) that has reciprocal interactions with co-chaperones and other HSPs. The HSP70 family members in various organelles (e.g., ER, mitochondria) do not contain the C-terminal EEVD motif; instead, these HSP70s have unique targeting signals that determine their localization [47,48].

The N-terminal NBD provides an ATP/ADP pocket for ATP binding that is of critical importance to the ATPase reaction required for folding and release of client proteins. The subdomain SBDβ is a peptide-binding pocket to which polypeptides could bind as substrates. The rapid association and timely dissociation of substrates are necessary for HSP70s to prevent peptide aggregation and perform substrate folding [49]. Mechanistically, the HSP70 ATPase cycle depends on an allosteric effect of both functional domains. More specifically, conformational changes of HSP70 molecules influence both the hydrolysis of ATP to ADP and the binding and release of substrates. Furthermore, ATP hydrolysis can increase the substrate affinity for SBDβ and promote substrate binding. On the other hand, the dissociation of ADP and the binding of new ATP increase the rate of substrate exchange and facilitate the release of substrates.

The functional cycle of HSP70s can be accelerated by a wide range of co-chaperones that primarily include (i) J-domain proteins (i.e., JDP family), such as HSP40, which are critical for the catalysis of ATP hydrolysis and increase in ATPase activity; (ii) nucleotide exchange factors (NEFs), such as Bag-1 and HSP110, which can accelerate the release of ADP (one of the crucial processes of HSP70 functional cycle); and (iii) TPR domain co-chaperones, such as Hop and CHIP, all of which are able to bind to the EEVD motif present in HSP70s and essential for assembly of the HSP70 complex. In addition, CHIP exhibits ubiquitin ligase activity and is consequently involved in the ubiquitination of some HSP client proteins.

### 3.1. HSP70 (HSC70 and Inducible HSP70)

The term HSP70 is a vague description. This term usually refers to both inducible HSP70 encoded by *HSPA1* and HSC70 encoded by *HSPA8* or to all HSP70 family members (e.g., HSP70s in this review). HSC70 is the major, noninducible cytosolic HSP70, while the stress-inducible form of HSP70 is the second most abundant cytosolic homolog. Despite the distinct difference in both HSP70 molecules in protein-coding genes and expression patterns, HSC70 is 86% homologous to stress-inducible HSP70 [47]. Analogous to other HSP70 family members, HSC70 and its inducible form are structurally composed of two functional domains, N-terminal NBD and C-terminal SBD, which are essential for their ATP-dependent cyclic function.

Mammalian HSC70 is encoded by the gene *HSPA8*. The *HSPA8* gene is constitutively expressed in most tissues. At the cellular level, its expression is observed mainly in the cytoplasm and also observed in the exosome and on the plasma membrane [50]. The gene *HSPA8* has been shown as one of the important housekeeping genes. Its knockout is lethal to cells, highlighting the critical role of the *HSPA8* gene in cell survival. Mammalian HSC70 performs its housekeeping functions to maintain cellular homeostasis, including folding of nascent polypeptides, transmembrane transport of proteins, prevention of protein accumulation under stress conditions and disassembly of clathrin-coated vesicles [51,52]. Additionally, one more event that HSC70 participates in is a special autophagy process called chaperone-mediated autophagy (CMA). The HSC70 degrades soluble cytosolic substrates by performing CMA and thus regulates cell cycle progression and initiates various diseases such as cancer [53]. The involvement of HSC70 in all these biological processes depends on its ability to shuttle proteins between cytoplasm and organelles. Furthermore, HSC70 has been shown to shuttle between cytoplasm and nucleus. This shuttle machinery confers its ability to facilitate import/export of different client proteins into/from the nucleus [54].

The inducible HSP70 virtually includes a wide range of chaperone proteins. This term is usually referred to as inducible HSP70-1a and HSP70-1b that are encoded by *HSPA1A* and *HSPA1B*, respectively, and these two inducible molecules are collectively called HSP70-1 (i.e., HSP72 or HSPA1). HSP72 is the primary member of the stress-inducible HSP70 family [47] and expressed in response to proteotoxic stress mediated by heat shock factor 1 (HSF1) [49]. Human HSP72 is primarily localized in the cytoplasm and nucleus, but it is also expressed at the lysosome. In addition, its membrane-anchored and secreted forms have been detected, especially in diseased conditions [54]. Apart from HSP72, there are other two inducible forms of HSP70 called HSP70-2 and HSP70-6 which are encoded by *HSPA2* and *HSPA6*, respectively. HSP70-2 and HSP70-1 differ by merely two amino acids, making them difficult to distinguish from each other by conventional experiments [54]. However, neither HSP70-2 nor HSP70-6 have been well studied until now. Thus, we will focus on inducible HSP70-1 (HSP72) in this review.

Within stressed cells, human HSP72 performs its cytoprotective function via (i) attenuating the proteotoxicity by interacting with stress-damaged cellular proteins and their aggregates [49] and (ii) inhibiting stressed cell death by the blocking apoptotic or necroptotic pathway and stabilizing inhibitor of apoptosis proteins (IAPs) [55,56,57]. The HSP72 molecule also regulates cellular signaling and protein degradation so as to maintain the viability of stressed cells including cancerous cells [55,56,58]. Furthermore, another study performed by Powers and colleagues shows that genetic reduction of *HSPA8* leads to increased expression of HSP72, which provides evidence for the interaction between two HSP70 molecules: HSC70 and HSP72 [59].

### 3.2. GRP78 (HSPA5)

The 78 kDa glucose-regulated protein GRP78, also known as HSPA5 or binding immunoglobulin protein (Bip), is an ER-resident molecular chaperone. Human GRP78 exists primarily within the ER where it is involved in the quality control of proteins, including folding, maturation, degradation, transport and secretion of proteins, and participates in the maintenance of intracellular Ca^2+^ homeostasis [60]. Under stressed conditions, GRP78 tends to increase and its upregulation prevents protein aggregation and facilitates degradation of misfolded proteins [61,62]. The GRP78 molecule serves as a master regulator of unfolded protein reaction (UPR) that is triggered by hypoxia, hypoglycemia, Ca^2+^ imbalance and other conditions. Within the unstressed cells, GRP78 is inactively bound to three ER-specific stress signal transducers (SSTs): activating transcription factor 6 (ATF6), inositol-requiring kinase 1 (IRE1) and dsRNA-activated protein kinase-like ER kinase (PERK). Once unfolded proteins accumulate within the ER, three SSTs dissociate from GRP78, and these liberated SSTs are able to regulate certain signal pathways and activate downstream effectors [63]. The products of ER stress-responsive genes include calreticulin, the component of ER-associated protein degradation (ERAD) serving for the proteolysis, and inducible GRPs (GRP170, GRP94, GRP78, GRP75) that catalyze either disaggregation or refolding of stress-damaged proteins within the ER and mitochondria in an ATP-dependent manner [17]. When accumulation of unfolded and misfolded proteins is reduced, GRP78 expression and its binding to three SSTs return to normal; however, once UPR fails to restore homeostasis, uncontrolled UPR leads to macroautophagy or cellular apoptosis [64,65].

Different from other HSP70 family members, GRP78 structurally contains a unique C-terminal KDEL sequence (KDEL retention motif) recognized by the KDEL receptor for molecular retrieval from the Golgi resulting in ER retention [66,67]. When GRP78 is upregulated within the ER, a subfraction of GRP78 escapes from the ER retention and translocates to the plasma membrane since KDEL receptors are fully saturated, and then GRP78 turns into cell surface GRP78 (csGRP78) [68]. csGRP78 exists predominantly as a peripheral protein on the plasma membrane with three different configurations: (i) as a membrane-embedded protein, (ii) associated with a transmembrane protein or (iii) bound to glycosylphosphatidylinositol (GPI)-anchored protein (e.g., Cripto, T-cadherin) [69]. This cell surface molecule serves as a multifunctional receptor for various ligands, such as α2-macroglobulin (α2M*) [70], some classes of viruses [71], plasminogen kringle 5 (K5) and major histocompatibility complex class I (MHC-I) [72]. csGRP78 molecule is also implicated in modulation of a variety of signal pathways as diverse as the PI3-kinase (PI3K)/AKT pathway, NF-κB-dependent pathways, the Ras/mitogen-activated protein kinase (MAPK) signal pathway and other pathways [73,74].

### 3.3. Mortalin (GRP75, HSPA9)

Mortalin, namely GRP75 or mitochondrial HSP75 (mtHSP75), is firstly found in the mitochondria. The gene *HSPA9* encodes this protein, which has 52% amino acid homology to inducible isoform HSP70 (HSP72) [47]. Mortalin contains functional domains NBD and SBD that are structurally homologous with other HSP70 family members; however, the C-terminal EEVD motif seems not to exist in its amino acid sequences. Furthermore, the presence of a 46-amino acid mitochondrial targeting sequence that confines mortalin within the mitochondria was reported by Dahlseid and colleagues in 1990s [75]. As a mitochondria-resident protein, mortalin is naturally associated with various physiological processes of mitochondria, such as maintenance of mitochondrial integrity, energy metabolism, free-radical generation and biogenesis [76]. This association may be due to the central role mortalin has in the import machinery of nuclear gene products in the mitochondria [77]. One of the specific functions of mortalin within mitochondria is to assist in protein quality control as a stress-survival factor by (re)folding or degrading nonfunctional proteins [78,79,80]. For example, mortalin assures the correct folding and proper assembly of intramitochondrial protein by cooperating with HSP60-HSP10 so as to maintain mitochondrial homeostasis under stress conditions [81]. As an essential molecule in the presequence translocase-associated motor (PAM) complex, mitochondrial mortalin also carries out the translocation of precursor proteins into the mitochondrial matrix by binding to these preproteins [82].

Although predominantly localized in the mitochondrial compartment, mammalian mortalin is also found in extramitochondrial sites when overexpressed, including the cytosol and the perinuclear region. This ATP-dependent chaperone is able to couple the inositol 1, 4, 5-triphosphate receptor (IP3R) on ER to the voltage-dependent anion channel (VDAC1) on mitochondria, which assists in Ca^2+^ transfer from the ER lumen to the mitochondrial matrix [83,84,85]. This coupling mechanism may be responsible for the maintenance of intracellular Ca^2+^ homeostasis mediated by mortalin. In the nucleus, human mortalin participates in the maintenance of telomere length, and it modulates genetic processes by controlling centrosome duplication in the course of chromosome replication and division, as well as mRNA processing and transport [86]. Furthermore, mortalin was shown to interact with onco-suppressor protein p53 in the perinuclear area, thereby repressing the expression of several p53 target genes, such as *CDKN1A* (encoding p21, cyclin-dependent kinase inhibitor 1), *MDM2* (an E3 ubiquitin-protein ligase) or *BAX* (an apoptosis regulator) [87,88,89,90]. The interaction of mortalin with p53 is responsible for the presence of several p53-mediated events such as cell cycle arrest, DNA break repair and apoptosis or senescence following genotoxic stresses [91]. Outside the cells, once stimulated by complement attack, secreted mortalin will translocate to the plasma membrane on which its ATP-binding domain binds to C5b-9 complex, thus preventing complement-mediated cell death [92,93,94,95].

## 4. The Association of HSP70s with BPH

### 4.1. HSP70s and Cell Survival, Proliferation and Apoptosis

The imbalance between proliferative rates and apoptotic rates of prostatic cells is one of the significant causes leading to BPH. As prosurvival factors, it has been noted that HSP70s facilitate cell survival by either stimulating cellular proliferation or inhibiting cell apoptosis in a number of diseases. In the presence of an HSP72 inhibitor, for example, PCa cell lines show an increase in the G1 phase and decrease in both the S phase and G2 phase (cell cycle arrest at G1 phase) and therefore show an inhibition of cell growth [96]. The GRP78 inhibitor rutaecarpine also arrests the cell cycle of prostate cancer cells at the G0/G1 phase [97]. Interestingly, we found in our published study that there was no significant difference in cell cycle distribution of cultured prostatic cells following GRP78 knockdown or overexpression versus normal controls [18]. We therefore made a conclusion that GRP78 may not initiate prostatic hyperplasia via modulating cell cycle progression. Moreover, GRP78 can bind to PI3K, the activator of AKT, to initiate the downstream AKT/mTOR signaling pathway and therefore to repress cellular apoptosis [98,99]. In agreement with these findings, we reported one way that GRP78 triggered prostatic hyperplasia was to upregulate the AKT/mTOR signaling pathway [18]. Our reports were strongly supported by the fact that AKT activator SC79 reversed the proapoptotic effects of GRP78 knockdown. The protective role of mortalin in apoptosis involves HIF-α. Mortalin has the ability to bind HIF-α so as to translocate HIF-α onto the outer mitochondrial membrane where HIF-α suppresses apoptotic under ERK inactivation [100]. However, few studies to date have focused on the role of mortalin in the pathogenesis of prostate diseases. With regard to the HSP72 molecule, apoptosis was shown to inversely correlate with HSP72 expression in PC-3 cells. Mechanistically, the HSP72 molecule prevents cell death not only through repressing cell apoptosis via inactivation of c-Jun N-terminal kinase (JNK), p38 and apoptosis-inducing factor (AIF) and reduced formation of death-inducing signaling complex (DISC) [101,102,103,104] but also through suppressing necrosis by inactivating JNK [105]. As for HSC70, Masako and colleagues reported that this protein prevented the degradation of Rab1A denatured by stress exposure and stabilized the Rab1A molecule, thus promoting cell survival [106]. This prosurvival effect of Rab1A arises from its ability to facilitate autophagosome formation and autophagy progression.

### 4.2. HSP70s and Oxidative Stress

Growing evidence has uncovered the relationship between HSP70 levels and oxidative stress status. An in vivo study using rodents observed that HSP72 overexpression reduced the release of ROS [107] and enhanced the activity of SOD [108]. In the prostate tissues resected with thulium laser from BPH patients, 70 kDa HSP72 can stimulate ROS generation and upregulate NOD-like receptor (NLR) to activate the ROS-NLRP3 signaling pathway, thereby inducing sterile inflammation in the prostate glands [20]. Mortalin has been reported to prevent the release of oxidant-induced cytochrome c from mitochondria [109]. This mitochondrial molecule has the ability to inhibit mitochondrial ROS production through stabilizing cytochrome c and other principal components of the electron transfer chain and/or through enhancing mitochondrial antioxidant mechanisms [110,111]. To date, no studies have investigated whether mortalin participates in blocking ROS formation in prostatic cells and prostate tissues. Our recently published work made it clear that genetic knockdown of GRP78 stimulated ROS production and decreased expression of antioxidant enzymes SOD and CAT in either BPH-1 or WPMY-1 cells [18]. In this study, we also elucidated that altered OS status induced with GRP78 silencing was attributed to inactivation of the AKT/mTOR pathway in the prostate.

### 4.3. HSP70s and EMT Process

It has been well accepted that HSP70 family members are able to activate the EMT process in various cancers and therefore facilitate cancer invasion and metastasis. Moreover, the aforementioned sections described EMT as a predisposing factor of BPH. There are few data, however, illustrating whether HSP70s are capable of triggering EMT events in the benign hyperplastic prostate. Our recently published work firstly reported that GRP78 induced the EMT process in cultured BPH-1 cells, as demonstrated by increased levels of N-cad and vimentin upon GRP78 overexpression [18]. Interestingly, we found in our study that overexpression of the *HSPA5* gene led to increased levels of E-cad expression. In most cases, the triggering of an EMT event is concomitant with downregulation of epithelial marker E-cad and upregulation of mesenchymal markers N-cad and vimentin. We further detected the levels of some EMT transcription factors (EMT-TFs), including Snail1, Snail2, Twist, ZEB1 and ZEB2, and found that siRNA-mediated GRP78 knockdown impressively increased Snail2 expression and decreased expression of other EMT-TFs. Since Snail2 has an inhibitory effect on E-cad expression in the prostate glands [112], we attributed the paradoxical upregulation of E-cad levels resulting from GRP78 overexpression to increased expression of its strong suppressor Snail2. As for other HSP70 family members, their molecular roles in EMT in the course of BPH are required to be further investigated in the future.

### 4.4. HSP70s and AR

**Figure 2:**
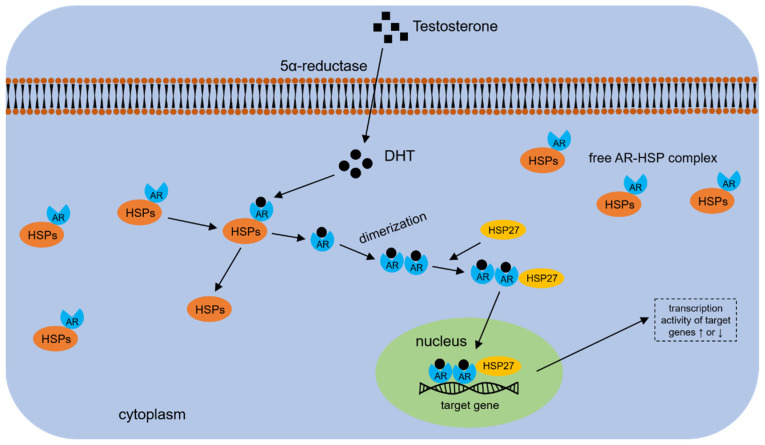
Schematic illustration of androgen-AR action in prostate cells. HSPs, including HSP70s and HSP90s, are in complexes with AR molecule in the cytoplasm. Testosterone is converted into DHT by 5α-reductase; this action allows HSPs to disassociate from the AR-HSPs complex and facilitates AR dimerization. The AR dimer then translocates into the nucleus and binds to the promoter regions of target genes by recognizing androgen response elements (AREs), thereby performing its various functions.

As early as the 1990s, HSP70s were isolated and purified from the AR heterocomplex in LNCaP cells, showing that HSP70s are components of the AR heterocomplex and appear to have interactions with AR in prostate cancer cells [113]. Similar to other steroid receptors, the ligand-free AR resides in the cytoplasm of prostatic cells and forms an AR-HSP complex with heat shock proteins (e.g., HSP40s, HSP70s), as well as their co-chaperones [114,115]. Binding of androgens to AR leads to dissociation of AR from HSPs and its translocation from the cytoplasm to the nucleus (Figure 2).

Among all HSP70 family members, HSP72 and GRP78 have been confirmed to be associated with AR in prostatic cells. On a molecular level, HSP72 has reciprocal interactions through its SBD with the N-terminal domain of AR [116]. Indirect evidence supporting this finding comes from a study of murine motor neuron hybrid cells reporting that HSP72 promoted degradation of expanded polyglutamine repeat AR by interacting with the N-terminal domain where the polyglutamine repeat resides [117]. The binding of HSP72 to AR in prostate glands influences endogenous AR levels, and targeting this interaction with the HSP72 inhibitor blocks AR signaling, leading to decreased transcript activity of AR [116]. Moses et al. also reported the presence of HSP72 in complexes with full-length AR (FL-AR) and ARv7 (one of the AR splicing variants) in prostate tissues and identified HSP72 as an important cofactor of AR that contributed to its stability and/or function [118]. On the other hand, DL3, an antagonist for AR signaling, was reported to downregulate HSP72 expression by reducing mRNA transcription with marginal effects on mRNA stability in human prostate cancer cells [119]. The ability of AR to positively regulate the *HSPA1* gene may arise from its occupation of the promoter region of *HSPA1*. This effect can be attenuated by DL3, which explains the inhibitory function of DL3 on HSP72 expression. In summary, HSP72 is able to regulate the expression level of AR; AR and its signaling in turn have a regulatory effect on HSP72 expression. This bidirectional crosstalk between two molecules may constitute a feedback mechanism to maintain the level of intracellular HSP72 in the prostate.

Analogous to the HSP72 molecule, GRP78 is also a critical AR-responsive gene in prostate glands. Data on prostate cancer tissues have shown that GRP78 expression in AR(+) tumors is significantly higher than in AR(−) tumors [120]. AR upregulates GRP78 expression and regulates ER stress response; consistently, DHT increases both AR and GRP78 levels. In contrast, sorafenib downregulates AR and its downstream molecule GRP78 [120,121]. In addition, AR can interact with GRP78 and form an AR-GRP78-associated protein complex, which was confirmed by immunoprecipitation experiments, and DHT treatment facilitates this in vivo interaction [122]. In turn, GRP78 affects AR expression levels and its functional status. siGRP78-mediated GRP78 knockdown promotes AR aggregate formation, while GRP78 overexpression leads to inhibition of AR protein ubiquitination and aggregation [122]. An immunohistochemistry experiment performed by Tan et al. demonstrated that AR levels significantly correlated with GRP78 levels (correlation coefficient > 0.3, *p* < 0.01) [120]. Together with the regulatory function of AR on GRP78, it has been conjectured that, similar to HSP72, there may exist a feedback loop between GRP78 and AR.

Notably, the association with AR has been shown for inducible HSP72 and GRP78 but, up to now, not for mortalin. As a member of the HSP70 family, it is still unknown whether mortalin expression has a link with AR expression or not. If so, is mortalin an upstream regulator or a downstream target of the AR gene? Additionally, there are no mechanistic studies about the interaction of HSP70s with the AR molecule in BPH. Again, all these problems are waiting to be studied and solved in the future.

## 5. HSP70s as Potential Therapeutic Targets for BPH

Currently, medical therapies for BPH include α-adrenergic blockers, 5α-reductase inhibitors, phosphodiesterase inhibitors (PDEIs) and dozens of plant extracts. The 5α-reductase inhibitor is the only treatment to reduce prostate volume, of which finasteride is the most widely used medicine due to its durability of efficacy and minimal adverse effects. Although all these oral medications are effective and acceptable, they are not always capable of preventing BPH progression, and surgical intervention may be necessary for about 30% of BPH patients. HSP70s have been recently considered as promising molecular targets that may be applied for the treatment of many diseases such as prostate cancer. The aforementioned relationship between the HSP70 family and multiple pathogenetic factors of BPH implies that HSP70s could also be discovered as potential therapeutic targets for BPH treatment.

Small molecular inhibitors against HSP70s include a wide range of agents that have been documented to target either the C-terminal substrate-binding domain (e.g., 2-phenylethynesulfonamide (PES)) [123] or the N-terminal ATP-binding domain (e.g., 15-deoxyspergualin (15-DSG), MKT-077) [124,125]. Mechanistically, molecular inhibitors binding to C-terminal SBD are capable of disrupting the association of HSP70s with their co-chaperone HSP40 and other client proteins, while those targeting the N-terminal ATP-binding domain are able to disrupt the ATPase activity of HSP70s, thereby both inhibitors can induce cell apoptosis and reduce the volume of target organs. All these molecular inhibitors have been well studied in many cancers, including prostate cancer [16,125,126], but most of them have not been successfully developed for commercial use, with only MKT-077 being clinically tested.

Monoclonal antibody (mAb) therapy against HSP70s may become one of the most promising therapeutic choices for BPH in the future. Compared with small molecular inhibitors, mAb targets HSP70s more accurately with fewer side effects due to its high antigen specificity. An antibody called cmHsp70.1 recognizes the extracellular motif TKDNNLLGRFELSG (TDK) of membrane-bound HSP70, and it induces antibody-dependent cellular cytotoxicity (ADCC) to kill target cells by binding the TDK motif [127]. In colon cancer, the cmHsp70.1 antibody has been reported to decrease the survival rate of cancer cells [128].

In addition to antibodies, the development of HSP70 vaccines, based on the immunogenic properties of HSP70s, offers a novel therapy for hyperplastic diseases. Several vaccines composed of disease-specific epitopes and HSP70 DNA are subjected to clinical trials, such as pNGVL4a-Sig/E7(detox)/HSP70 DNA [129]. This HSP70 vaccine in the body can stimulate and activate CD8+ T cells. Activated T cells are differentiated into cytotoxic T lymphocytes (CTLs) and exert their functions of killing target cells specifically. However, neither HSP70 antibodies nor HSP70 vaccines have been studied in prostate diseases. In fact, the cytotoxicity of all the above-mentioned agents confers their ability to induce cell death of prostate cells. Thus, the development of these agents against HSP70s is clinically useful for the treatment of hyperplastic diseases such as BPH.

However, all aforementioned agents have such a powerful proapoptotic effect that their side effects may not be acceptable when used for oral or intravenous delivery for BPH treatment. Furthermore, a small- to medium-sized prostate gland is characterized by a predominance of fibrosis, indicating that antifibrotic therapies are more effective than proapoptotic therapies for men with small- to medium-sized prostates. Only for large prostate glands, therapies to decrease the prostate size may become one of the important choices. Therefore, a great number of studies are required to determine the mode of administration of the above-mentioned agents (intraprostatic injection), as well as the objects treated with these medications. If these scientific problems can be solved, the HSP70 family will become a promising therapeutic target for BPH.

## Data Availability

Not applicable.
